# Portable LiDAR-Based Method for Improvement of Grass Height Measurement Accuracy: Comparison with SfM Methods

**DOI:** 10.3390/s20174809

**Published:** 2020-08-26

**Authors:** Hiroyuki Obanawa, Rena Yoshitoshi, Nariyasu Watanabe, Seiichi Sakanoue

**Affiliations:** 1 Hokkaido Agricultural Research Center, National Agriculture and Food Research Organization (NARO), Sapporo 062-8555, Japan; saka@affrc.go.jp; 2 Western Region Agricultural Research Center, National Agriculture and Food Research Organization (NARO), Oda 694-0013, Japan; r_yoshitoshi@affrc.go.jp (R.Y.); nariyasu@affrc.go.jp (N.W.)

**Keywords:** light detection and ranging, structure from motion, grass height, point cloud, digital surface model

## Abstract

Plant height is a key indicator of grass growth. However, its accurate measurement at high spatial density with a conventional ruler is time-consuming and costly. We estimated grass height with high accuracy and speed using the structure from motion (SfM) and portable light detection and ranging (LiDAR) systems. The shapes of leaf tip surface and ground in grassland were determined by unmanned aerial vehicle (UAV)-SfM, pole camera-SfM, and hand-held LiDAR, before and after grass harvesting. Grass height was most accurately estimated using the difference between the maximum value of the point cloud before harvesting, and the minimum value of the point cloud after harvesting, when converting from the point cloud to digital surface model (DSM). We confirmed that the grass height estimation accuracy was the highest in DSM, with a resolution of 50–100 mm for SfM and 20 mm for LiDAR, when the grass width was 10 mm. We also found that the error of the estimated value by LiDAR was about half of that by SfM. As a result, we evaluated the influence of the data conversion method (from point cloud to DSM), and the measurement method on the accuracy of grass height measurement, using SfM and LiDAR.

## 1. Introduction

In order to increase forage yield and quality in grassland farming, it is necessary to decide the appropriate harvest time based on accurate growth assessment. Grass height, which is an important indicator of growth conditions, has been measured by various methods. The simplest, most economical and frequently used method is direct measurement with a ruler. However, it is time-consuming, and is not practicable for detailed plant height distribution with high-density measurement.

In recent years, the structure from motion and multi view stereo (SfM-MVS) photogrammetric method has been successfully used to estimate the height and biomass of plants. For example, Cooper et al. [1] carried out digital camera photography from eye height level, on 11 grass plots in South Dakota, USA, and showed that biomass could be estimated from the volume of the grass material reconstructed by SfM. Researchers have also used this method to determine tree height [2,3], wheat height [4], pasture height [5,6,7,8], and reconstruct three-dimensional structures of legumes and carrots [9]. Vegetation classification can also be performed, using aerial images captured by an unmanned aerial vehicle (UAV) [10,11]. However, plant measurement using SfM has limitations, e.g., difficulties in reconstructing fine shapes, such as branches in three dimensions [2], insufficient reconstruction of canopy top [1], and inability to use on windy days [1,9].

Light detection and ranging (LiDAR) is among the most advanced and promising sensing methods. It measures distance using the time taken by a laser beam to reflect off an object and return (time of flight method). As the laser irradiation frequency is very high (generally several hundred thousand shots/second), it becomes possible to acquire detailed point cloud data with high spatial resolution [12,13]. By changing the rotation speed of the sensor’s motor, the density of the point cloud can be adjusted. LiDAR has been successfully applied for the measurement of plants [14]. For example, Kaizu [15] estimated pasture height for a grassland in Hokkaido, Japan, using LiDAR mounted on a tripod at a height of 3 m, and reported that the RMS error with the actual grass height was 7.4 cm. Hosoi and Omasa [16] reconstructed a detailed plant structure, based on the vertical distribution of plant area density of paddy rice. Radtke et al. [17] reported that the contour lines of crown measured by LiDAR have different shapes depending on the grass species. Other researchers have also used LiDAR to measure the height of vegetables [18], pasture height [19], corn height [20], and biomass of Arctic shrubs [21]. As mentioned by Cooper et al. [1], LiDAR is predominantly used to estimate forest biomass, and more rarely, for grasslands, due to its poor ability to measure plant structure in windy conditions. However, even if the object sways in the wind, LiDAR may be able to measure the canopy height of the grass, whereas SfM cannot measure it using the principles of photogrammetry. That is, it is possible to measure the shape of the target object at the moment when the laser is irradiated. It is considered that the shape of the grass group can be obtained by measuring the same place multiple times, even in a short time. Additionally, with the prices of LiDAR equipment dropping sharply in recent years, it is expected to become an effective and affordable tool for measuring the shape of agricultural crops in the future, by using it in combination with UAVs.

In this study, we aimed to verify the effectiveness of LiDAR in plant measurement. We measured grass using both SfM and LiDAR, and compared the accuracy of estimates of plant heights by each method. We particularly considered the creation of the digital surface model (DSM) of the surface of grass-canopy, and the ground used for grass height calculation. We also examined the influence of the conversion method from 3D point cloud acquired by SfM or LiDAR to two dimensional DSM, and the spatial resolution of the DSM on the estimation accuracy.

## 2. Materials and Methods

### 2.1. Study Site

**Figure 1:**
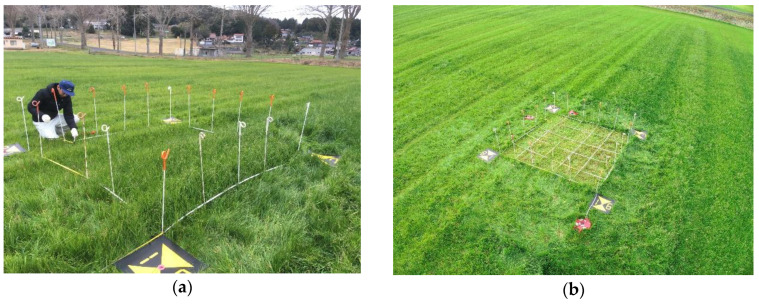
Study site: Italian ryegrass (*Lolium multiflorum* Lam.) grassland (**a**) before harvesting and (**b**) after harvesting.

For this study, we selected an almost flat 2.5 m × 2.5 m section of an Italian ryegrass (*Lolium multiflorum* Lam.) grassland with approximately 10-mm wide leaves at Oda Research Station, Western Region Agricultural Research Center, National Agriculture and Food Research Organization, Japan (Figure 1, 35°10′2.864″ N, 132°30′19.669″ E, WGS84). Ground markers of 50 cm square were placed on the four edges of the section for alignment with other measurement data. We subdivided the target area into 25 smaller plots of about 50 cm × 50 cm, and plant height was estimated and compared for each small plot. In general, spatial variation in plant height tends to be significant. As such, it is desirable to maintain a small measurement range, to obtain an accurate and representative value using a conventional ruler. Herein, the estimation accuracy of the true grass height was improved by reducing the measurement range to a plot size of 50 × 50 cm. The measurements were carried out on 6 January 2020, 14:30–16:30 JST, under cloudy weather, and an average wind speed of 0.9–2.0 m/s (Automated Meteorological Data Acquisition System (AMeDAS) Oda: 2.6 km north-northwest of the survey site, approx. 9 m higher than the survey site).

### 2.2. Measurement Procedures

**Figure 2:**
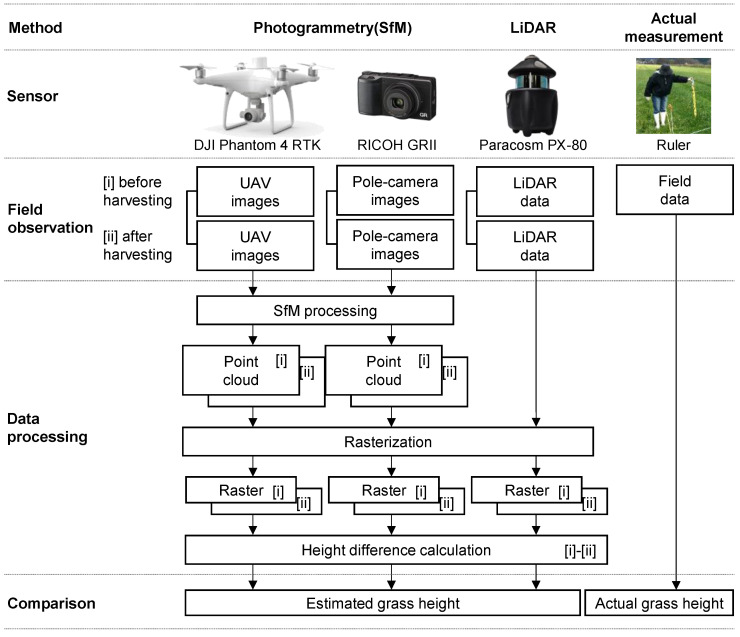
Outline of the measurement procedure.

We followed the experimental procedures in the following sequence (see Figure 2 for an outline and the following sections for details): (1) We performed aerial photography using UAV and pseudo aerial photography from a height using a pole camera. (2) We conducted measurements using a hand-held LiDAR. (3) We measured the grass height using a ruler. (4) The grass was harvested at 3 cm from the ground. (5) We repeated steps 1 and 2 after harvesting.

### 2.3. UAV Aerial Photography

**Figure 3:**
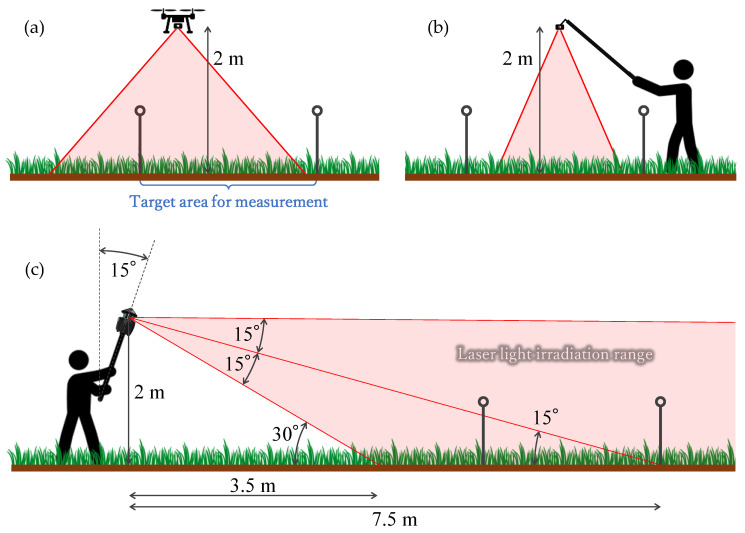
Schematic diagram of measurements: (**a**) unmanned aerial vehicle (UAV); (**b**) pole camera; and (**c**) light detection and ranging (LiDAR).

The UAV used for aerial photography (Phantom 4 RTK, DJI, Shenzhen, China) was capable of virtual reference station (VRS) positioning by a built-in global navigation satellite system (GNSS) receiver. Hence, it was possible to assign highly accurate position coordinates to each aerial photograph: 1 cm + 1 ppm (horizontal) and 1.5 cm + 1 ppm (vertical), based on the manufacture’s spec sheet. Photography was performed in a vertically downward direction, from an extremely low altitude (about 2 m above the ground) that was similar to the height for LiDAR measurement described below (Figure 3a). Despite windy conditions, the displacement of the UAV position in flight, shake of the shooting direction of the camera apparatus, and camera shake effects were hardly recognized due to the high-precision positioning by RTK-GNSS, and stabilization provided by the gimbal system mounted on UAV.

### 2.4. Pole Camera Photography from a Height

Since the UAV aerial photography was carried out from an extremely low altitude (about 2 m), we were apprehensive that the swaying of grass caused by the downdraft from the UAV propeller might adversely affect the subsequent SfM processing. Therefore, we attached a digital camera (GRII, RICOH, Tokyo, Japan) to the tip of the pole, and the target area was photographed from above, as a pseudo aerial photography method free from downdraft. Photography was carried out about 2 m height above the ground, in accordance with the LiDAR measurement (Figure 3b). The shooting direction was diagonally downward because the camera angle was manually adjusted by the operator while moving.

### 2.5. LiDAR Measurements

We carried out LiDAR measurements using a hand-held scanner (PX-80, Paracosm, Gainesville, FL, USA). This scanner had a measurable distance range of 1–100 m, and could measure about 300,000 points per second (not changeable), with a field of view 360° (horizontal) and 30° (vertical). It was cylindrical in shape (26.4 cm high, 16.2 cm diameter), and weighed about 3 kg. The maximum scan time with the built-in battery was about 40 min. Hence, it could be operated as a hand-held LiDAR. The integrated point cloud data were output by built-in simultaneous localization and mapping technology, that simultaneously estimates the position of the moving body, and integrates point cloud data to create a surrounding environment map. For measuring the shape of the target area, we attached the scanner body to the end of the pole, holding it with both hands, and raised it to a height of about 2 m above the ground. We tilted it at a zenith angle of about 15°, and rotated it from a position about 5 m away from the target area for taking measurements (Figure 3c). Thus, the incident angle of the laser light in the measurement range was about 20° in elevation from the horizontal. The distance from the hand-held LiDAR to the measured target equaled approximately 7 m, with the beam size on the ground of 31.8 mm for the horizontal and 20.0 mm for the vertical directions.

### 2.6. Measurement by a Ruler

We measured the grass height using a ruler at 5 points in each small plot, which were averaged to obtain the actual value of grass-canopy height. Enumeration was carried out by a single grassland survey expert consistently, to prevent bias in the readings.

### 2.7. Post-Processing

**Figure 4:**
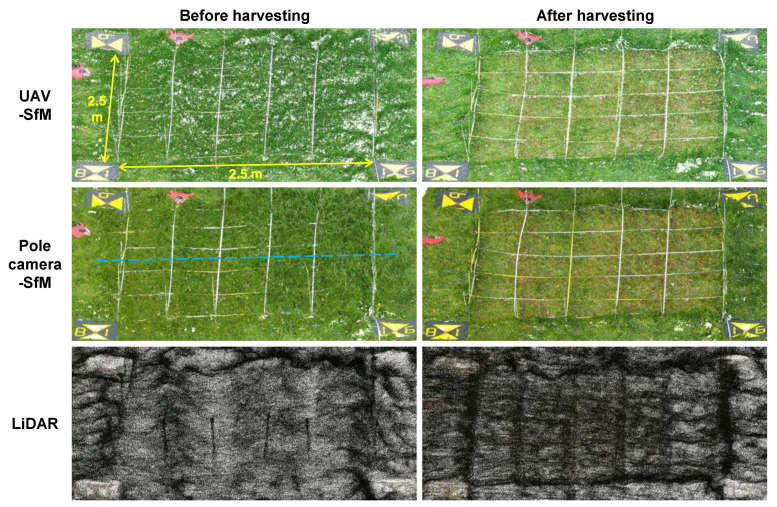
Bird’s-eye view of the point clouds. The blue dashed line represents the survey line of sections presented in Figure 5.

The photogrammetric data collected from the UAV and the pole camera were analyzed for SfM-MVS processing, using the Agisoft Metashape Pro. Ver.1.5.2 software to generate 3D point cloud data (Figure 4). For UAV-SfM, the coordinates recorded by the VRS method by the GNSS receiver mounted on the UAV at the instant of shooting were used as position information for the 3D point cloud data. Regarding the 3D point cloud data for the pole camera-SfM and LiDAR, the coordinates of the ground markers were read from the 3D model created by UAV-SfM, and the position coordinates were assigned as their ground control points. Hence, following the aforementioned process, 3D point cloud data were achieved through three different methods—UAV-SfM, pole camera-SfM, and LiDAR. The point cloud density (average of two measurements before and after harvesting) was 789 points/cm^2^ for UAV-SfM, 269 points/cm^2^ for pole camera-SfM, and 9 points/cm^2^ for LiDAR.

## 3. Results and Discussion

### 3.1. Comparison of Point Cloud Data

**Figure 5:**
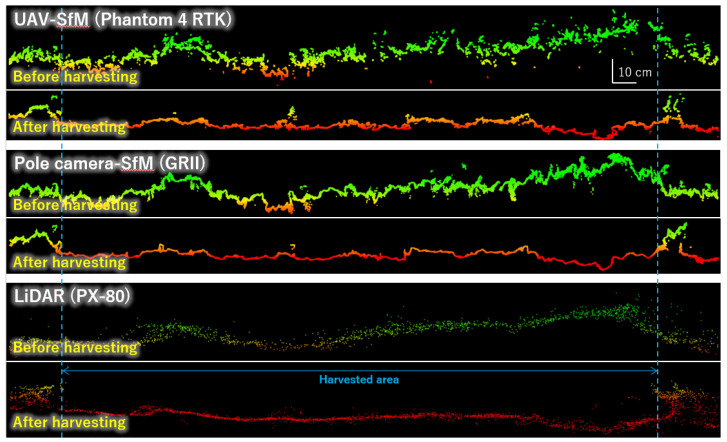
Section views created from the point cloud data. The survey line runs approximately east-west along the center of the measurement range, as shown in Figure 4. Point cloud within a width of 5 mm along the survey line is projected horizontally.

We examined the cross sections of the point cloud data (Figure 5). Regarding the shape before harvesting, the cross section from UAV-SfM showed unevenness, the pole camera-SfM had some unevenness, while the cross section from LiDAR was quite smooth. This was possibly because the point cloud density was UAV-SfM > pole camera-SfM >> LiDAR, and the large number of point clouds likely affected the resolution of fine irregularities in the cross section. Moreover, in the case of UAV-SfM, since the grass leaves were subject to shaking by the downdraft generated by UAV, a large amount of noise was possibly generated during the SfM processing process, resulting in increased irregularities in the cross-sectional view. Conversely, with regards to LiDAR, it was possible that the shape of the cross-sectional view was smoothed, because the leaves swaying by the prevailing wind were measured multiple times with laser light during enumeration. The cross-section shape after harvesting, however, showed almost no difference between UAV-SfM and pole camera-SfM, and the cross section of LiDAR was quite smooth. As above, this was likely because the LiDAR data had a lower point density than those from the other two SfM methods; therefore, a fine shape was not reproduced. Another possible reason for this could be that SfM mainly depicted the surface shape of the short grass remaining after harvesting, while LiDAR mainly reconstructed the shape of the ground surface by the laser beam that passed between the grass leaves.

### 3.2. Conversion Method for Point Cloud to DSM

To estimate plant height, we created DSMs before and after harvesting. In data conversion, we investigated the influence of the selection method of height data in each DSM cell, and the spatial resolution of DSM on the accuracy of grass height estimation. From the three methods, we obtained representative height data (i.e., maximum, mean, and minimum values) from the point cloud contained in each DSM cell. We employed four selection methods (Table 1): (1) maximum selection method, i.e., the difference between the maximum height before harvesting and after harvesting, (2) average selection method, i.e., the difference between the average height before and after harvesting, (3) minimum selection method, i.e., the difference between the minimum height before and after harvesting, and (4) maximum-minimum selection method, i.e., the difference between the maximum height before harvesting and the minimum height after harvesting. DSM spatial resolution was set to seven cell sizes—5, 10, 20, 30, 40, 50, and 100 mm.

**Figure 6:**
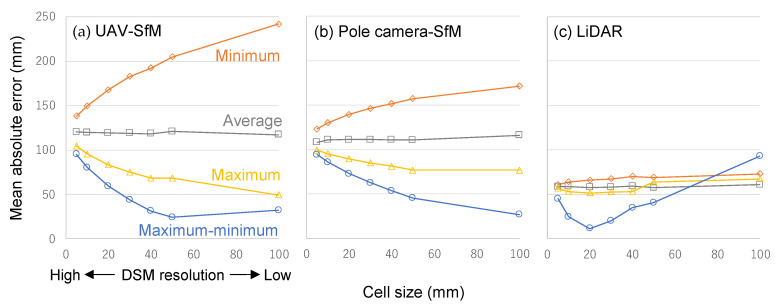
Effects of conversion method from point cloud data to height data of DSM cell, and DSM resolution on accuracy of plant height estimation: (**a**) shows the results of the UAV- structure from motion (SfM) method; (**b**) shows the results of the pole camera-SfM method; (**c**) shows the results of the LiDAR method.

We compared the results from each method (Figure 6). The value of grass height recorded using the ruler was assumed to be the true value. To avoid the possibility of the data being mixed up due to the collapse of grass between adjacent small plots (50 cm square), we set the comparison range to 30 cm square at the center of each small plot. Overall, the UAV-SfM method showed the largest variation and error value (24–242 mm), due to the height selection method and DSM resolution. The pole camera-SfM method gave a moderate variation (27–172 mm), while LiDAR returned the smallest variation and error (12–93 mm). For the two SfM methods, the order of error values (in decreasing order of magnitude) for different selection methods was as follows: minimum > average > maximum > maximum-minimum. The height data selection method had a significant influence on the error. For instance, under the UAV-SfM method and DSM resolution of 50 mm, errors in the minimum selection method and the maximum-minimum selection method were 205 mm and 24 mm, respectively, which differed by a factor of about nine. Even for the average selection method, the error was 121 mm (about 5 times the error in the maximum-minimum method). In the average selection method, there was negligible change with the spatial resolution of DSM. We compared our results to the previously published ones. For example, Batistoti et al. [7] estimated the height of pasture by UAV photogrammetry, and reported that the average error from the actual measured value was 80 mm. Viljanen et al. [5] reported that the difference between the grass heights estimated by UAV aerial photogrammetry and the actual measured value was 64–122 mm (RMS error). Although the previously reported error values are similar, the accuracy of the estimated values in our study was rather low, considering the imaging height differences (2 m in this paper vs. 50 m in Batistoti et al. [7] vs. 30–50 m in Viljanen et al. [5]) The SfM method accuracy was almost certainly affected by the camera quality, weather conditions, density of vegetation, height of grass, and the shooting altitude. As such, we recommend further considerations for the effects of each factor.

Conversely, for the LiDAR method, the error values were nearly constant under minimum, average, and maximum methods (52–73 mm). Here, the error for maximum-minimum selection method was relatively small, except for the DSM resolution of 100 mm. Specifically, the error was the smallest (12 mm) when the DSM spatial resolution was 20 mm, and increased sharply at resolutions smaller or larger than that. Hence, the optimal data processing conditions for improving the accuracy of grass height estimation were as follows: the maximum-minimum selection method for height data for both SfM and LiDAR methods and a DSM spatial resolution of 50–100 mm for the SfM method, and 20 mm for LiDAR method.

A possible reason for the maximum-minimum selection method giving the least error was that the maximum value in the point cloud represented the surface shape of the leaf tip most accurately, and the minimum value represented the ground shape most accurately. Therefore, the maximum-minimum selection method accurately represented the grass height, i.e., the difference in vertical height between the surface shape of the grass-canopy and the ground. We considered the following possible explanations for the effect of the spatial resolution of DSM on the grass height estimation error. SfM, which is a photogrammetric method, could not accurately reconstruct the three-dimensional shape of the leaf tip of the grass swaying by the downward airflow generated by UAV, or the prevailing wind during measurement before harvesting, and hence could not sufficiently express the fine unevenness of the leaf tip surface. Furthermore, since the number of point clouds that could accurately express the leaf tip height was relatively small, when the DSM resolution was high (small cell size), the leaf tip height was underestimated. Conversely, when the DSM resolution was low (larger cell size), the leaf tip height could be expressed more accurately. For measurement after harvesting, using SfM made it difficult to assess the ground shape three-dimensionally, which was likely to be obscured by short grass. Therefore, the number of points that could accurately reconstruct the ground height was relatively small, and at lower DSM resolution, the ground height could be expressed more accurately. As a result, for the SfM method, lowering the spatial resolution of DSM improved the measurement accuracy.

In contrast, LiDAR, a laser-based survey, could more accurately represent the surface shape of the leaf material, by measuring the same location multiple times, even when the leaf tip was shaking due to wind. Additionally, LiDAR could represent the ground shape more accurately, as the laser could pass between the leaves. Therefore, when the width of the target leaf was about 10 mm, the coarser DSM possibly could not express the unevenness of the leaf tip surface sufficiently with the maximum value before harvesting. Moreover, the finer DSM could not sufficiently express the ground shape, as the minimum value captured the shape of the residual leaf blade after harvesting. The fact that the actual beam size of LiDAR was approximately 20–30 mm (refer to Section 2.5) might have also affected the optimum cell size. As a result, a resolution of about 20 mm represented the actual plant height (the height of the leaf tip relative to the ground) more accurately. Cooper et al. [1] reported that, for both SfM and LiDAR methods, the DSM resolution did not affect the estimation accuracy of plant height when the DSM resolution was 2 cm or more. We found that, in both SfM and LiDAR methods, the plant height estimation error did not depend on the DSM resolution under the average selection method (Figure 6). Therefore, it appears that their findings were due to the use of averages when converting point clouds to DSM.

Generally, measurement accuracy is assumed to improve with increasing DSM resolution (smaller cell size). However, we observed that the error increased when the DSM resolution was high for all methods. Hence, both SfM and LiDAR methods require careful attention to the optimum resolution when creating DSM from point cloud data.

### 3.3. Comparison between SfM and LiDAR

**Figure 7:**
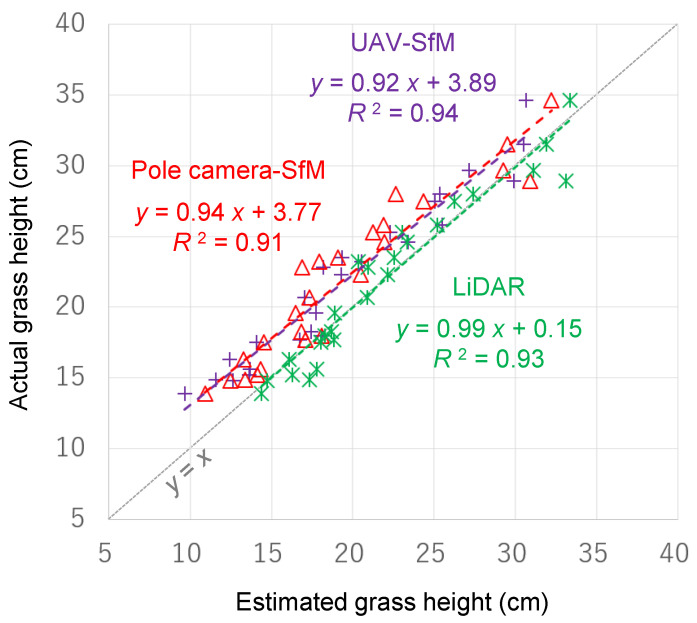
Comparing the grass height values estimated by SfM and LiDAR with the actual values measured by a ruler. The gray dashed line represents the 1:1 line for reference.

We compared the plant height values estimated by SfM and LiDAR with the height measured by a ruler (Figure 7). The conversion method from point cloud data to DSM was set so as to minimize the error in each method. Hence, for height, we used the maximum-minimum selection method for SfM (both UAV and pole camera), as well as LiDAR, while the DSM resolution was 50 mm, 100 mm, and 20 mm for UAV-SfM, pole camera-SfM, and LiDAR, respectively. As a result, the average absolute errors (mean ± standard deviation) were 24 ± 13 mm, 27 ± 15 mm, and 12 ± 10 mm, for UAV-SfM, pole camera-SfM, and LiDAR, respectively. The error value of LiDAR was significantly lower than those of both UAV-SfM and pole camera-SfM (*p* < 0.01, one-way ANOVA followed by Tukey’s multiple comparison test using the R statistical package, version 3.6.3 [22]). Our results indicated that the error in the estimated value by the LiDAR method was about half compared to that for the SfM methods, and that LiDAR method estimates were closest to the true values. The reason why LiDAR performed better than SfM at measuring grass height was likely due to its higher capability for reconstructing the surface shape of swaying grasses and the ground between grass leaves (see Section 3.2). The relationship between the size of the grass (height) and the margin of error was unclear. Furthermore, the correlation coefficients between the estimated values and the measured values were 0.95 or more for all three methods, which suggests that actual values can be derived from estimated values by using an appropriate calibration formula. However, the calibration formula of the SfM-estimated value is likely to change with the grass species and the growth stage. Hence, the LiDAR method, which provided estimates closest to the actual measured value without calibration, is better for measuring the plant height directly and more accurately.

### 3.4. Future Outlook for SfM and LiDAR

UAV-SfM can be installed and operated at a lower cost than LiDAR. In addition, further improvements in UAV performance (e.g., improved flight time, loadable capacity, weather resistance, and RTK-GNSS performance), camera performance (e.g., better resolution, sensor size), and PC performance (e.g., shorter image processing time) will enable a wider range of measurements at higher speed in the future. However, because it is based on photogrammetric technology, UAV-SfM presents several disadvantages, such as being unable to measure plants height in the wind, or the ground shape that is slightly visible between the leaves. On the contrary, LiDAR devices are typically expensive, presenting a constraint for their practical application. However, with rapid technological advancement and price reduction, LiDAR will likely gain widespread usage with effortless application in agriculture. With the LiDAR devices becoming lighter and more compact, we expect them to be more commonly used with consumer-grade light-weight UAV, making it possible to measure wider areas.

As Vázquez-Arellano et al. [13] mentioned, both SfM and LiDAR have disadvantages and advantages; therefore, complementary sensor fusion could facilitate a more robust performance. For example, in the livestock sector, it is almost impossible at present to accurately measure the amount of foraging by cattle during grazing. An examination of changes in plant height with UAV-SfM generally includes an error of more than 10 cm, which is indistinguishable from food intake. If UAV-LiDAR can measure the amount of foraging with high accuracy, and UAV-SfM can accurately assess the distribution of grass species, it will be possible to define the type and amount of grass eaten by cattle. That is, precision farming, including advanced cattle management and optimization of feeding, will be made possible. In this light, we believe that LiDAR has immense potential to become a key technique in agricultural remote sensing.

## 4. Conclusions

When converting height information from the point cloud to DSM for grass height measurement by SfM and LiDAR methods, we found that accuracy was maximized by using the maximum value of the point cloud before harvesting, and the minimum value of the point cloud after harvesting as data for each DSM cell. Moreover, for a leaf width of about 10 mm, the spatial resolution of DSM that gave the highest accuracy of estimated grass height was 50–100 mm for SfM and 20 mm for LiDAR. Particularly for LiDAR, we found that the measurement error increased when the DSM resolution was too large or too small. Moreover, comparing the performance of SfM and LiDAR for grass height measurement, we found that LiDAR estimated a value closest to the actual value determined by a ruler, with the error reduced to about half. These findings are potentially useful in choosing a method for grass height measurement, selecting post-processing methods for the acquired data, and evaluating the accuracy of the processed data. However, the versatility and generality of the methods presented herein have to be examined further in various environments in the future.

## Figures and Tables

**Table 1:** Acquisition method of height data in each cell of digital surface model (DSM) from point cloud.

Method	Selection Method of DSM Height in Each Cell from Point Cloud	
	Before Harvesting	After Harvesting
Maximum	Maximum height	Maximum height
Average	Average height	Average height
Minimum	Minimum height	Minimum height
Maximum-minimum	Maximum height	Minimum height
